# Are Lockdowns Effective in Managing Pandemics?

**DOI:** 10.3390/ijerph19159295

**Published:** 2022-07-29

**Authors:** Moshe Yanovskiy, Yehoshua Socol

**Affiliations:** Department of Electrical and Electronics Engineering, Jerusalem College of Technology, Jerusalem 9116001, Israel; mosheya@jct.ac.il

**Keywords:** COVID-19, Spanish Flu, disaster management, decision making, health and wealth

## Abstract

The present coronavirus crisis caused a major worldwide disruption which has not been experienced for decades. The lockdown-based crisis management was implemented by nearly all the countries, and studies confirming lockdown effectiveness can be found alongside the studies questioning it. In this work, we performed a narrative review of the works studying the above effectiveness, as well as the historic experience of previous pandemics and risk-benefit analysis based on the connection of health and wealth. Our aim was to learn lessons and analyze ways to improve the management of similar events in the future. The comparative analysis of different countries showed that the assumption of lockdowns’ effectiveness cannot be supported by evidence—neither regarding the present COVID-19 pandemic, nor regarding the 1918–1920 Spanish Flu and other less-severe pandemics in the past. The price tag of lockdowns in terms of public health is high: by using the known connection between health and wealth, we estimate that lockdowns may claim 20 times more life years than they save. It is suggested therefore that a thorough cost-benefit analysis should be performed before imposing any lockdown for either COVID-19 or any future pandemic.

## 1. Introduction

The present coronavirus crisis caused major worldwide disruption which has not been experienced for decades. The word ‘lockdown’, originally meaning “*the confinement of prisoners to their cells for all or most of the day as a temporary security measure*” according to Webster’s dictionary [1] emerged with a new meaning: Schools and workplaces closed, closure or restrictions on dining, sports, and cultural events, extraordinary travel restrictions, canceled medical and dental visits, curfews, quarantine regulations and more [2,3]. The lockdown-based crisis management was implemented by nearly all the countries, and studies confirming lockdown effectiveness can be found alongside with the studies questioning it.

Our aim in this work was to learn lessons and analyze ways to improve the management of similar events in the future. To achieve this, we have performed a narrative review of the works studying the above effectiveness, as well as the historic experience of previous pandemics. Moreover, we aimed to perform a cost-benefit analysis to compare lockdowns’ benefits (lives saved) with cost (lives lost). The following research questions have been formulated: What has been known about lockdowns’ effectiveness in saving/prolonging life in previous pandemics; what is the COVID-19 evidence regarding lockdowns’ effectiveness in saving/prolonging life; which factors determine the human cost of lockdowns—side-effects that shorten life; how can the human cost of lockdowns be estimated based on economical parameters; what is the quantitative estimation for lockdowns’ human cost? Finally, we tried to understand how decision-making was actually performed. The research questions are listed in Table 1.

## 2. Methods

There have been published numerous papers on various aspects of the COVID-19 crisis: for example, PubMed^®^ (pubmed.gov) collection alone lists 93,478 papers published in 2020 and 138,429 in 2021. Due to the vast amount of relevant literature, we found it unfeasible to perform a systematic review of the existing literature. Instead, we performed a narrative review following an iterative procedure discussed below.

**Figure 1:**
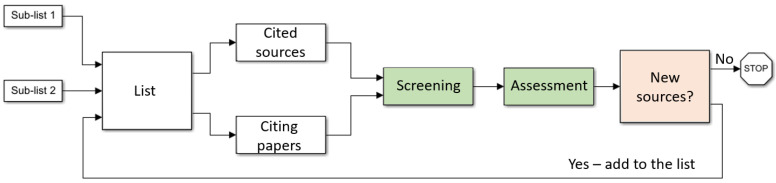
Block diagram of the performed search. Sub-list 1: all COVID-19 papers published in 2020 in the highest-impact journals in the fields of medicine and economics. Sub-list 2: pre-2020 publications of selected authors directly related to the COVID-19 crisis management. Screening: initial screening performed by the first author (MY). Assessment: final assessment performed by both authors. New sources: cited sources and citing papers leftover after the two-stage screening.

First, an initial list (described in the next paragraph) of a limited number of sources (entries) was produced. Then, papers cited in the latter entries were reviewed, as well as subsequent works citing the entries. Since we did not perform a systematic review, no rigorous inclusion or exclusion criteria were defined. Instead, we performed a subjective assessment whether the new entry either provided considerable new information or cited important sources related to the research questions. Regarding previous pandemics and the connection between health and wealth, works conducted after 9/11 (when the topic of emergency management became extensively funded) were subjected to additional scrutiny to exclude possible bias. The suitability of references was double-checked by a two-stage screening process: first screening by the first author (MY) with random control by the second author (YS), followed by a final assessment by both authors. Entries that passed the screening were added to the list. The above process was iterated several times, until no new sources providing considerable information were found. The process is illustrated in Figure 1. Since the search converged, we believe that the choice of initial sources was not crucial.

The initial list for the iterative search was compiled from two sub-lists. The first sub-list contained all COVID-19 papers published in 2020 in the highest-impact journals in the fields of medicine (*Nature*, *Science*, *The Lancet*, *The New England Journal of Medicine*) and economics (*Journal of Economic Literature*, *Journal of Political Economy*). The second contained selected papers of authors of important (often pioneering) works, published before 2020 but directly related to the COVID-19 crisis management: Gary Becker, Daron Acemoglu—health and economic growth; Kip Viscusi—value of statistical life (VLS) indicator to measure policy effectiveness; John Ioannidis—epidemiology and population health; Christian Bjørnskov—economic history.

Modern economic growth, observed at least since the Industrial Revolution, has been accompanied by impressive growth in trade, travel, and population density. At the same time, in recent centuries there have been no examples of pandemics that killed a large part of the population in many countries.

The focus of this small study is on established democracies because in the rest of the world there are significant statistical reliability problems encountered. Also, in authoritarian countries, the motivation for decision-making is noticeably different.

As a result of the described survey, a collection of about two hundred works (referred to as “the Collection” below) was compiled [4]. Then, we performed an evidence-based risk-benefit evaluation of the lockdowns’ effectiveness. Analyzing decision-making, we exploited one of the basic assumptions of the Public Choice theory stating the absence of an ideal omniscient interest-free government [5].

## 3. Results

### 3.1. Lockdowns’ Effectiveness in Controlling Pandemics

#### 3.1.1. Previous Pandemics

Influenza-like pandemics are a natural consequence of human development [6]. Therefore, they should not be considered a global threat. The history of the 1918 H1N1 influenza type-A pandemic (the Spanish Flu) and numerous less-severe pandemics is well documented—see references in sect. II of the Collection [1]. Analysis of this well-documented history shows that the COVID-19 problems are not new, unlike round-the-globe governmental reactions that are unprecedented and definitely not based on any successful policy in the past.

The quite reasonable idea (from the point of view of contagion mechanisms) of the effectiveness of social distancing during epidemics is not supported by vast evidence. Somewhat, on the contrary, modern economic growth is associated with an unprecedented increase in travel (partly related to trade) and a significant increase in population density as most of the population becomes urban rather than rural. That is, the average social distance has become much less. At the same time, in recent centuries there were no examples of epidemics that killed a quarter to a half of the population in dozens of countries, such as with the Justinian Plague [7] or Black Death [8].

The Spanish Flu was one of the deadliest pandemics in centuries, and for sure the most lethal of well-documented pandemics [9]. The highest mortality was detected among the 20–40 years age group [10] and so caused sizable demographic damage—unlike COVID-19. This being said, there was no panic then and the response was mainly based on common sense—see references in sect. II.2 of the Collection [4]. The leaders generally presumed reasonable and rational behavior of the citizens: e.g., quarantine practices were almost exclusively voluntary. Closure of the non-entertainment business was out of discussion [11]. School closure was discussed but often rejected due to the obvious outcome: children’s bands roaming in dirty streets could boost the infection spread more than gathering in supervised and relatively clean schools [12].

The effectiveness of various closures has not been proven. For example, mortality rates were similar in New York (535 per 100,000) [12] and Los Angeles (494 per 100,000) [11], even though in Los Angeles schools, churches, and places of entertainment were closed for up to 6 weeks [11], and in New York, everything remained open [12]. It should be noted that New York was a port city with a mass return of troops—infected by the flu—from Europe.

Two extensively cited papers [13,14] argue that government intervention and coercion are justified by the Spanish flu pandemic’s experience of suppressing mortality. The work claims that in cities that imposed earlier and harsher restrictions, both the mortality was lower, and the after-flu economic recovery was faster.

The benefits of coercive social distancing are justified using the indicator of authorities’ coercion (Non-pharmaceutical Interventions—NPI) in the 42 largest cities in the United States from 1918–1919. The “NPI intensity” is defined as the duration of the imposed restrictions, completely ignoring their nature and extent. Essentially, the authors assume that the restrictions in all 42 cities of the sample were the same. Therefore, New York falls into a subsample of cities with strong intervention (total NPI days—73 days, above the median)—despite the fact that schools and theaters were not closed there.

The principal “intervention” in New York was staggered business hours to avoid rush hour in the public transit system, agreed with big business and approved by city authorities [12]. This intervention caused minimal compliance costs for businesses and was insignificant even compared with pretty moderate NPI in the rest of American big cities during the Spanish Flu.

On the opposite, Pittsburgh (the most severely-affected city of the sample) is classified by the cited authors as unacceptably “liberal” with 52 NPI days (below median)—though during these 52 days theaters and schools were closed.

The Pittsburgh case is a clear example of the authors’ failure to include a significant local factor in the model. Namely, the high mortality rate in Pittsburgh has a good explanation not connected to the alleged oversight and ‘liberalism’ of the authorities. Even before the Spanish flu, many more people (compared to the US average) died in Pittsburgh from respiratory diseases due to severe air contamination by metallurgical plants’ emissions [15].

#### 3.1.2. Preparedness Plans

It should be mentioned that the same conclusions—no clear benefit of lockdowns in case of pandemic—were made by national and international bodies before COVID-19 emerged. Namely, several governments prepared detailed plans of response to influenza-like pandemics years ago—see the programs of the U.S. Occupational Safety and Health Administration (2007) [16], Israeli Ministry of Health (2007) [17] and more references in sect. II.4 of the Collection [4]. Israel probably had the most elaborate plan which relied on the unique experience of civil-military partnership (ref. II.4.4 in the Collection [4]). Noteworthy, the World Health Organization (WHO) published its comprehensive 91-page preparedness plan [6] in October 2019!

All these response plans mentioned lockdowns, if at all, as a means of last resort only. The WHO document, for example, explicitly mentioned that:

- social distancing measures “can be highly disruptive” and should be carefully weighted
- travel-related measures are “unlikely to be successful”; “border closures may be considered only by small island nations in severe pandemics”
- contact tracing and quarantine of exposed individuals are not recommended in any circumstances.

All the above plans were abandoned without any serious discussion at the very beginning of the COVID-19 crisis: The authors failed to find a single mention of these plans in publications of national health ministries in either country. Lockdowns, border closures, contact tracing, and quarantines became the main instruments.

#### 3.1.3. COVID-19

We saw that pre-COVID-19 evidence was against lockdowns that were nevertheless imposed in nearly every country. A posteriori, the pro-lockdown evidence, while extensively cited, was based largely on comparing real-world outcomes against computer-generated forecasts derived from models that were not tested empirically, such as that of Flaxman et al. [18] or Brauner et al. [19]. A recent review analyzed the 1st lockdown (Spring 2020) in 130 countries and, after making multiple adjustments, concluded that early school and workplace closures saved around 1.5 daily deaths per million [20] (we shall discuss later the implications of this number).

On the other hand, comparative studies of different countries showed no connection between the stringency of response measures (non-pharmaceutical interventions) and actual outcomes. For example, the American Institute for Economic Research prepared in December 2020 a digest [21] of 35 works showing no such connection; see also refs. [22,23] and more references in sect. III.3 of the Collection [4]. Later research (January 2022) performed at Johns Hopkins University [24] also concluded that “lockdowns have had little to no effect on COVID-19 mortality”.

On the contrary, the lockdown policies had a direct side effect of increasing mortality. Hospitals in Europe and USA were prepared to manage pretty small groups of highly contagious patients, while unprepared for a much more probable challenge—large-scale contagion. As a result, public health care facilities and nursing homes often became vehicles of contamination themselves—to a large extent because of the lockdown-based emergency policy implementation, e.g., New York’s policy of forcing nursing homes to admit recently discharged COVID-19 patients; see references in sect. IV.1 of the Collection [4].

### 3.2. Human Cost of Lockdowns

#### 3.2.1. Factors of Life Loss Due to Lockdowns

Numerous deaths can be attributed to the interruption of normal social life and routine regular social interactions. The direct factors are [25,26]:

- increased mortality due to postponement of diagnoses and routine treatments
- increase in mortality due to non-arrival at hospitals
- increase in mortality due to a decrease in the level of income and as a result—use of less safe cars, reduction in the scope of physical activity, etc.
- “deaths of despair” caused by drugs, alcohol, and suicide following loss of social-economic status
- increase in violence, including domestic violence; dismantling of families
- severe health damage to the elderly in particular—physical and mental deterioration (usually irreversible) due to loneliness, lack of movement, and routine supportive care.

More information can be found in sect. IV.2 of the Collection [4] and on the *Collateral Global* charity website (CollateralGlobal.org).

While it is very difficult to quantify lockdowns’ negative effects on public health with precision, one can make rough estimations based on economic losses and the connection of health and wealth. This is conducted in the following subsections.

#### 3.2.2. Health and Wealth

Irrefutable historical facts are the following: modern progress in life expectancy, health status, and sharp decrease in infant mortality—all followed the economic progress and were clearly explainable by economic progress. ‘Modern economic growth’ manifests in per capita growth, rather than just population growth, which is the only reliable indicator of economic progress during the pre-capitalist epochs. Modern economic growth caused drastic improvements in housing conditions, food, clean water, and sanitation [27,28]. In turn, a drastic decrease in morbidity and early death encouraged more investment in human capital [29]. The rise in education and income prompted demand for healthcare services, and eventually also for medical research. Advanced healthcare and vaccination contributed back in stronger incentives to invest in health and education, producing a virtuous cycle [30].

Since increased income led to life extension, it is logical to conclude that lost income means lost lives. Corresponding references can be found in sect. I.3 of the Collection [4]. Therefore, any decision potentially harmful to personal income should be scrutinized, and decision-makers should not ignore the loss of life caused by loss of income and corresponding loss of socioeconomic status and self-respect [31,32].

Some authors argue on the above connection between health and wealth. Some of them point out that the positive trend of mortality decrease was not altered during the Great Depression, therefore questioning the negative health effects of the economic factors—see refs. [33,34] and more in section I.4. of the Collection [4].

All these authors generally ignore long-term trends. Since the beginning of the 20th century (at least), the overall mortality decreased and life expectancy increased, mainly due to the decrease in infectious diseases’ mortality. The overall mortality decrease was accompanied, however, by the increase in cardiovascular and cancer mortality: Roughly speaking, more and more people die of heart attack or cancer being 50–60 years old because they did not die of diphtheria or tuberculosis at age 30–40. However, during the Great Depression, the cardiovascular and cancer mortality growth rate slightly increased compared to both pre-Depression (1909–1929) and post-Depression (1940–1960) periods. Cardiovascular diseases and cancer are linked to psychosomatic consequences of job loss etc. (while any psychosomatic factors in carcinogenesis are a matter of debate, there is no doubt that psychosomatic factors affect a person’s resistance to illness and reaction to treatment). While the overall mortality continued to decrease during the Depression, the slope of this decrease certainly did not grow despite extensive government health programs which were a part of the New Deal.

In summary, it can be said: Though the evidence of the Great Depression’s negative effect on health is inconclusive, the opposite claims—of the overall positive effect of the massive government involvement in health during that period—are certainly not backed by the existing data.

Some researchers have even made a bold claim that the lockdowns coupled with economic recession could cause improvements in public health. We suggest the reader to compare this claim made for the broad audience via a popular resource [35] with the in-depth study *by the same authors* providing evidence for just the opposite [32]: All-cause mortality among the industrial workers who lost their jobs in the 1970s–1980s and never regained their socio-economic status, demonstrates an increase in the 2000s–2010s in stark contrast to the general trend of declining mortality in the developed countries.

Among the key sources that should be cited here is judicial commentary on an expensive regulation of Occupational Safety and Health Administration, in which Judge Williams of the D.C. Circuit stated that excessive regulatory expenditures would make society poorer, potentially worsening individual health. This opinion probably urged the Office of Management and Budget to raise the issue of the potentially counterproductive effects of excessive regulation [36].

#### 3.2.3. Cost-Effectiveness Threshold

Cost-effectiveness analysis is routinely performed in health policy. It is widely assumed (though far not unanimously) that the state should not provide citizens with services (including life-extension services) that are less cost-effective than the citizens themselves are willing to pay for such services [37]. Very different values for the cost-effectiveness threshold (CET) can be found in the literature. In our opinion, the proper approach to calculate CET is to use ‘willingness to pay’ (WTP)—that is, how people themselves value their lives in monetary terms [38]; the term WTP itself describes most exactly the mechanism behind the assumption that the state should not provide citizens with services costing above CET. We are going to discuss this mechanism now.

Accepting certain or very probable death for money is morally unacceptable. However, taking small risks for monetary compensation is routine. Each occupation is associated with some risk. Some occupations are riskier than others (firefighters, police, and even actors). If on average people are ready to take risk of death with a probability, e.g., of 0.001 (one of a thousand) for $1500, then WTP should be estimated as $1.5 million. Namely, to obtain $1.5 million in total wealth, a thousand people on average will take the risk 0.001 to die, and one on average will die. Though people take their risks voluntarily, the net effect is that public expenditure of one WTP statistically claims one human life. The above method of valuing life was proposed by Adam Smith more than two centuries ago and has been used since then in economic analysis as well as in legal practice [39]. Therefore, CET is not about the monetary value of life. It is about extending the life of the entire population.

#### 3.2.4. Human Cost of Lockdowns—Quantitatively

In the case of the COVID-19 crisis management, the extent of human life lost due to lockdowns can be roughly estimated based on the value of about 150% GDP per capita per quality-adjusted life-year (QALY) as the upper limit of prudent expenditure on healthcare and safety [40]. Yanovskiy et al. [41] quantified the human life loss in Israel: The total cost of lockdowns during the year 01.04.2020–31.03.2021 was estimated as about US$ 30 billion based on (a) the data of Bank of Israel and (b) the Oxford COVID-19 Government Response Tracker; while the Israeli population was about 9.2 million, and GDP per capita—about US $45,000. By dividing 30 billion by 1.5 × 45,000, the estimation of 500,000 QALY lost to lockdowns was obtained.

Another comparison can be made if we remember that the average age of people dying of COVID-19 was around 80, with 3–6 QALY per death lost. Therefore, 500,000 QALY are equivalent to roughly 100,000 COVID-19 deaths. Even if we assume that lockdowns saved 1.5 daily deaths per million [20] for a whole year (365 days), after multiplying by 9.2 million (population of Israel) we arrive at about 5000 lives saved—just about 5% of the lockdowns’ human cost. In other words, it can be estimated that even if the lockdowns saved some lives, in the long term they killed 20 times more.

To put the above number of 500,000 QALY into proportion, such life loss was found to be the equivalent of life years lost in Israel to cancer for 4 years [41]. Probably, in other developed countries, the human cost of lockdowns was also comparable with several years of life lost to cancer. The latter hypothesis is based on the fact that lockdowns imposed in Israel were of medium strictness according to the Oxford COVID-19 Government Response Tracker (OxCGRT) prepared by the Blavatnik School of Government [2]. In addition, Israel’s population density and national wealth are also rather typical for developed countries.

### 3.3. Decision Making

A thorough analysis of the lockdown decision-making is beyond the scope of this paper. However, it seems to us important to raise several questions.

- As mentioned above, the prepared response plans, both national and international, were abandoned without any serious discussion at the very beginning of the COVID-19 crisis. The actual response consisted of instruments that had been considered ineffective and counter-productive.
- The extent of human life loss has probably never been calculated and has never been taken into consideration in the decision-making process. One of the first reports recommending strict lockdowns (26 March 2020) explicitly stated [42]: “we do not consider the wider social and economic costs of suppression, which will be high”. Anyhow, societies have never been informed about these considerations and calculations.
- The implemented policy relied on compulsion instead of compassion and private initiative (with very few exemptions). The governments ignored alternative ways to protect groups at risk—see Sect. V.3 of the Collection [4].
- The forecasts which were chosen for political decision-making systematically overestimated the COVID-19 threat, supporting excessive measures—see references in sect. III.1. of the Collection [4]. Political leaders and government officials systematically “instilled fear in the population, thereby contributing to the making of mass hysteria” [43].
- The evidence-based approach was actually abandoned, as blatantly reasoned in the pro-lockdown open letter to the UK Chief Medical Officers [44]:
  *“Whilst it is always helpful to have more data and more evidence, we caution that in this complex and fast-moving pandemic, certainty is likely to remain elusive. ‘Facts’ will be differently valued and differently interpreted by different experts and different interest groups. A research finding that is declared ‘best evidence’ or ‘robust evidence’ by one expert will be considered marginal or flawed by another expert.”*

- Lockdowns were still imposed during the subsequent waves (autumn/winter 2020/21 and 2021/22, to say nothing about the 2022 spring lockdowns in PRC [45]) as if they had been proven effective—despite the above-mentioned evidence.

## 4. Discussion

It is correct that our understanding of viral transmission mechanisms leads to the assumption that lockdowns should be an effective pandemic management tool if long-term collateral damage is neglected. However, the *post factum* analysis yields the opposite result. Many factors could contribute to the lack of lockdowns’ effectiveness; consideration of these factors lies far beyond the scope of this paper. We just mention the mechanism of aerosol transmission [46] and the low percolation threshold for contagion in the modern densely interconnected society [47].

The extreme measures that deprived billions of their basic human rights followed (without any reasonable discussion) the abandonment of well-prepared crisis management plans. The extent of human life lost due to lockdowns themselves has never been quantitatively reported and therefore never been taken into consideration in the decision-making process. Moreover, governments continuously stuck to these measures despite the absence of proof that such measures were effective in controlling the pandemic. Nor the Italian government, the first among democratic countries to impose a nationwide lockdown, neither the authorities in other countries published materials showing how the known negative consequences of lockdowns were taken into account when making a decision (which could prove *a posteriori* to be correct or erroneous) that the expected gains outweigh the losses. Publication of the results of the scientific analysis would certainly strengthen public support of the authorities and their decisions. The absence of such publications probably means that such analysis was not performed.

We should stress here that the burden of proof is with the lockdown proponents. Lockdown opponents do not have to prove that lockdowns cause damage, the proponents must prove that lockdowns are beneficial. The latter statement follows from the two basic principles, which are outlined below.

The first is the classical medical principle ‘*primum non nocere*’—first, do not harm. The meaning of this principle is that the fear of ultimately harming by intervention should clearly prevail over the fear of not helping (while nearly every medical procedure, surely every surgery, is associated with some harm). This principle is valid no matter how serious the medical problem is; it should be valid for public health as well. The harm caused by lockdowns was obvious a priori and confirmed a posteriori—unlike the benefits of these interventions, as discussed above.

The second foundation is the classical juridical principle ‘*semper necessitas probandi incumbit ei qui agit*’—in any dispute, the burden of proof lies with those who lay charges. A citizen does not lay charges against the government; the government lays charges against citizens—to wear masks, to close their business, to stay at home.

The precautionary principle (PP) is sometimes cited to defend costly governmental interventions without solid scientific justification. However, even some PP supporters agree that in the case of governmental responses to COVID-19 pandemics (lockdowns and mandatory vaccination) even lowered standards of scientific justification, required by PP, were not met [48,49].

Such behavior—shifting the burden of proof to the opponents—provides ground for speculations that the decisions were not made exclusively on a professional and interest-free basis. Special interests of the decision-making groups provide partial explanations of the unprecedented policy.

Even in democratic countries with limited and accountable governments, decisions are made not by angels but by humans (even if they are elected representatives of government officials) with their own characters, biases and interests [50,51,52]. The special interests could well be altruistic if decision-makers were sincerely sure that their activity was in the best interest of the society; in addition, the experience of the PRC in controlling the epidemic by emergency measures—impressive in real time though questionable *a posteriori*—undoubtedly biased the early decisions made all over the world.

However, decisions on lockdowns may have also been politically motivated—see sect. V.5 of the Collection [4]. During the COVID-19 crisis, governments in general and public healthcare officials, in particular, enjoyed unprecedented expansion of power—to close schools and universities, send people to self-isolation, issue stay-at-home orders (*de facto*—house arrest without a court order), and more [2,3]. The expansion of funding was also unprecedented: the US Coronavirus Aid, Relief and Economy Security Act (“CARES Act”) alone was estimated to cost taxpayers $2.3 trillion (around 11% of GDP) [53] to be re-distributed by the government. The latter aspect of power and funding expansion could have contributed to the decisions to stick to the PRC pattern (ignoring the experience of countries such as South Korea, Taiwan, etc.) while abandoning the prepared plans and the evidence-based approach.

Moreover, even if decision-makers are interest-free, they cannot be ideal error-proof decision-making machines. One should not wonder that practical implementation of lockdowns often resulted in probably unexpected and surely undesired effects such as panic, increase in social tension and hostility, artificial crowding caused by document checks, etc.

One should not overlook the expansion of the limits of governmental power with a simultaneous decrease in accountability during the pandemic—see sect. IV.1, V.4, V.5, VI.1 of the Collection [4]. Abandonment of “wider social and economic costs” consideration de facto means disregarding the harm inflicted on personal liberties and democratic institutions by lockdown policies [54].

The questions of to what extent, why, and how the dissenting (disapproved by healthcare officials) scientific opinions were suppressed during COVID-19 [55] deserve a special and urgent analysis. Suppression of “misleading” opinions causes not only grave consequences for scientists’ moral compass; it prevents the scientific community from correcting mistakes and jeopardizes (with a good reason) public trust in science. At least, publicly funded research should be scrutinized for conflict of interest to avoid artificial scientific consensus [56].

Finally, it should be mentioned that even if hypothetically rigorous cost-benefit analysis in terms of the human cost would favor lockdowns, the very idea of saving the lives of people on account of the lives of others raises serious philosophical and ethical questions [26,57].

Our study is not free from limitations. The main limitation probably is that our resources did not enable performing a systematic literature review. Another important limitation stems from the probable bias in publications [56] mentioned above. Moreover, our study was based mainly on democratic countries with higher transparency. We anticipate that these issues will be addressed in detail by many future researchers. In addition, as time elapses, we anticipate long-term (hopefully lifespan) studies of the effects of both COVID-19 disease and lockdowns.

## 5. Conclusions

While our understanding of viral transmission mechanisms leads to the assumption that lockdowns may be an effective pandemic management tool, this assumption cannot be supported by the evidence-based analysis of the present COVID-19 pandemic, as well as of the 1918–1920 H1N1 influenza type-A pandemic (the Spanish Flu) and numerous less-severe pandemics in the past. The price tag of lockdowns in terms of public health is high: we estimate that, even if somewhat effective in preventing death caused by infection, lockdowns may claim 20 times more life than they save. It is suggested therefore that a thorough cost-benefit analysis should be performed before imposing any lockdown in the future. Our conclusions are summarized in Table 2.

## Figures and Tables

**Table 1:** Research questions of the present study.

	Research Question
1	What has been known about lockdowns’ effectiveness in saving/prolonging life in previous pandemics?
2	What is the COVID-19 evidence regarding lockdowns’ effectiveness in saving/prolonging life?
3	Which factors determine the human cost of lockdowns—side-effects that shorten life?
4	How can the human cost of lockdowns be estimated based on economical parameters?
5	What is the quantitative estimation for lockdowns’ human cost?
6	How was decision-making actually performed?

**Table 2:** Conclusions.

	Conclusions
1	Neither previous pandemics nor COVID-19 provide clear evidence that lockdowns help to prevent death in pandemic
2	Lockdowns are associated with a considerable human cost. Even if somewhat effective in preventing COVID-19 death, they probably cause far more extensive (an order of magnitude or more) loss of life
3	A thorough risk-benefit analysis must be performed before imposing any lockdown in future

## Data Availability

Not applicable.
